# Docosahexaenoic Acid Induces Apoptosis in MCF-7 Cells *In Vitro* and *In Vivo* via Reactive Oxygen Species Formation and Caspase 8 Activation

**DOI:** 10.1371/journal.pone.0010296

**Published:** 2010-04-22

**Authors:** Ki Sung Kang, Pan Wang, Noriko Yamabe, Masayuki Fukui, Taylor Jay, Bao Ting Zhu

**Affiliations:** Department of Pharmacology, Toxicology and Therapeutics, University of Kansas Medical Center, Kansas City, Kansas, United States of America; Bauer Research Foundation, United States of America

## Abstract

**Background:**

The present study sought to further investigate the *in vitro* and *in vivo* anticancer effects of a representative omega-3 fatty acid, docosahexaenoic acid (DHA), with a focus on assessing the induction of oxidative stress and apoptosis as an important mechanism for its anticancer actions.

**Methodology/Principal Findings:**

*In vitro* studies showed that DHA strongly reduces the viability and DNA synthesis of MCF-7 human breast cancer cells in culture, and also promotes cell death via apoptosis. Mechanistically, accumulation of reactive oxygen species and activation of caspase 8 contribute critically to the induction of apoptotic cell death. Co-presence of antioxidants or selective inhibition or knockdown of caspase 8 each effectively abrogates the cytotoxic effect of DHA. Using athymic nude mice as an *in vivo* model, we found that feeding animals the 5% fish oil-supplemented diet for 6 weeks significantly reduces the growth of MCF-7 human breast cancer cells *in vivo* through inhibition of cancer cell proliferation as well as promotion of cell death. Using 3-nitrotyrosine as a parameter, we confirmed that the fish oil-supplemented diet significantly increases oxidative stress in tumor cells *in vivo*. Analysis of fatty acid content in plasma and tissues showed that feeding animals a 5% fish oil diet increases the levels of DHA and eicosapentaenoic acid in both normal and tumorous mammary tissues by 329% and 300%, respectively.

**Conclusions/Significance:**

DHA can strongly induce apoptosis in human MCF-7 breast cancer cells both *in vitro* and *in vivo*. The induction of apoptosis in these cells is selectively mediated via caspase 8 activation. These observations call for further studies to assess the effectiveness of fish oil as a dietary supplement in the prevention and treatment of human breast cancer.

## Introduction

Omega-3 fatty acids (FAs^¶^) are long-chain polyunsaturated FAs. The principal dietary source of eicosapentaenoic acid (EPA, 20:5*n*-3) and docosahexaenoic acid (DHA, 22:6*n*-3) is from oily cold-water fish [1], [2]. Epidemiological studies have suggested that an increased fish oil intake is associated with a reduced breast cancer incidence in humans [3], [4]. Consistent with this epidemiological observation, laboratory studies have also shown that omega-3 FAs can suppress the formation and growth of breast cancer in animal models [5]–[8].

A number of mechanisms have been proposed for the anticancer actions of omega-3 FAs, including suppression of neoplastic transformation, inhibition of cell proliferation, enhancement of apoptosis, and antiangiogenicity [1], [9]. Most of these mechanisms have been directly or indirectly linked to their inhibition of the production of eicosanoids from omega-6 FAs [7], [9], [10]. In addition, it has also been suggested that increased lipid peroxidation may contribute to the anticancer actions of omega-3 FAs. Earlier studies have shown that accumulation of polyunsaturated FAs would lead to increased formation of lipid hydroperoxides as well as other degradation products that may be deleterious to cells [11]–[14]. Moreover, it was observed that the presence of antioxidants diminished their anticancer activity in cultured cancer cells, whereas the presence of prooxidants enhanced their anticancer activity [15], [16].

Using MCF-7 human breast cancer cells as an *in vitro* model, early studies have shown that omega-3 FAs not only inhibit their growth, but they also induce differentiation and apoptosis [17]–[19]. In the present study, we sought to further investigate the anticancer efficacy of omega-3 FAs, DHA in particular, on the growth of MCF-7 human breast cancer cells *in vitro* and *in vivo* (as xenografts in athymic nude mice), with a focus on assessing the induction of oxidative stress and apoptosis as a crucial mechanistic element for their anticancer activity. In addition, we have also determined the levels of omega-3 FAs in normal and tumorous tissues in athymic nude mice fed a 5% fish oil-supplemented diet. We believe a better understanding of the mechanism of the anticancer actions of omega-3 FAs would aid in the development of new cancer therapeutic strategies involving the use of fish oil as a dietary supplement.

## Materials and Methods

### Chemicals and reagents

DHA, EPA, linoleic acid (LA, 18:2*n*-6), arachidonic acid (AA, 20:4*n*-6), boron trifluoride, Eagle's modified minimum essential medium (EMEM, phenol red-free), and fetal bovine serum (FBS) were obtained from Sigma Chemical Co. (St. Louis, MO). Iscove's Modified Dubecco's Medium (IMEM) and an antibiotics solution containing 10,000 U/mL penicillin and 10 mg/mL streptomycin were obtained from Invitrogen (Carlsbad, CA). The trypsin-EDTA mixture (containing 0.25% trypsin and 0.02% EDTA) was obtained from Lonza Walkersville (Walkersville, MD). Caspase inhibitors (zVAD-FMK, IETD-FMK, LEHD-FMK, and zVEID-FMK) were obtained from EMD Chemicals Inc. (Gibbstown, NJ). Scramble siRNA and caspase 8-specific siRNA were obtained from Santa Cruz Biotechnology, Inc. (Santa Cruz, CA). All other reagents used in this study were obtained from standard suppliers and were of analytical grade or higher.

### *In vitro* experiments

#### Cell culture

MCF-7, MDA-MB-231 and MDA-MB-435s cell lines were obtained from the American Type Culture Collection (ATCC, Rockville, MD). MCF-7 cells were maintained in EMEM supplemented with 10% (*v/v*) fetal bovine serum, and MDA-MB-231 and MDA-MB-435s cells were maintained in IMEM (with 10% fetal bovine serum). These cells were incubated at 37°C under 5% CO_2_, and usually sub-cultured once every 3 days.

#### Assay of cell viability and DNA synthesis

For determining the rate of cell viability, the MTT [3-(4,5-dimethylthiazol-2-yl)-2,5-diphenyltetrazolium bromide] assay was used. MCF-7, MDA-MB-231 and MDA-MB-435s cells were seeded in 96-well plates at a density of 2,000 cells per well. The stock solution of DHA or EPA (100 mM in 200-proof ethanol) was diluted in ethanol first and then in the culture medium immediately before addition into each well at the desired final concentrations, and the treatment usually lasted for 3 days unless noted otherwise. For MTT assay, 20 µL MTT (at 5 mg/mL) was added to each well at a final concentration of 500 µg/mL, the mixture was further incubated for 1 h, and the liquid in the wells was removed thereafter. Fifty microliters of dimethyl sulfoxide were then added to each well, and the absorbance was read with a UV max microplate reader (Molecular Device, Palo Alto, CA) at 560 nm. The relative cell viability was expressed as a percentage of the control that was not treated with DHA or EPA.

The thymidine incorporation assay was used for determining the rate of DNA synthesis. Cells (in 96 well plates) were incubated in the presence of [methyl-^3^H]thymidine (5 nM, 1 µCi per well, PerkinElmer, Boston, MA) for 12 h. Then the cells were harvested onto glass fiber filters, and the incorporated radioactivity was counted using a β-scintillation counter (MicroBeta Trilux, PerkinElmer Life Sciences).

#### Flow cytometric analyses of cell death

The annexin-V and PI double staining method was used. MCF-7 cells were harvested with trypsin-EDTA and then stained with the Annexin V-FITC Apoptosis Detection Kit (BD Biosciences, San Jose, CA) according to manufacturer's protocols. Then the cells were subjected to flow cytometric analysis with a flow cytometer (model BD LSR II, BD Bioscience, San Jose, CA). In this assay, annexin-V would detect the translocation of phosphatidylserine from the inner leaflets to the outer leaflets of the plasma membrane, which is a key feature of apoptotic cells, whereas PI would detect necrotic cells with permeabilized plasma membrane.

#### TUNEL staining

Apoptotic DNA degradation was stained using the terminal deoxynucleotidyl transferase (TdT)-mediated dUDP-biotin nick end labeling (TUNEL) method. In this study, the ApopTag® Plus Peroxidase *In Situ* Apoptosis Detection Kit (Chemicon International, Temecula, CA) was used for this purpose.

#### Intracellular ROS measurement

For determination of the intracellular accumulation of ROS, the 2′,7′-dichlorofluorescein diacetate (H_2_-DCF-DA) method was used. After treatment of cultured cells (in 6-well plates) with DHA for the length of time as indicated, H_2_-DCF-DA (at a final concentration of 2 µM in the whole medium) was added to each well. After incubation for 20 min at 37°C, cells were washed with PBS twice with 20 min each time. Intracellular ROS accumulation was observed and photographed using a fluorescence microscope (AXIO, Carl Zeiss Corporation, Germany).

3-Nitrotyrosine level in cultured cells was determined using the immunocytochemical staining method. The specific antibody for 3-nitrotyrosine (1∶1000 dilution) was obtained from Cell Signaling Technology (Danvers, MA). Slides were counter-stained for nuclei with DAPI.

#### Transfection of siRNA and Western blotting analysis

siRNA was transfected into the cells using Lipofectamine 2000 (invitrogen) according to manufacturers' instructions. After 24 h, cells were harvested and washed with PBS, and then they were suspended in 100 µl of the lysis buffer (20 mM Tris-HCl, 150 mM NaCl, 1 mM EDTA, 1% Triton X-100, and a protease inhibitor cocktail, pH 7.5). The amount of proteins was determined using the Bio-Rad protein assay (Bio-Rad, Hercules, CA). An equal amount of proteins was loaded in each lane, separated by 12% SDS-polyacrylamide gel electrophoresis (SDS-PAGE), and electrically transferred to a polyvinylidene difluoride membrane (Bio-Rad). After blocking the membrane using 5% skim milk, target proteins were immunodetected using specific antibodies. All primary antibodies were obtained from Cell Signaling Technology (Beverly, MA). Thereafter, the horseradish peroxidase (HRP)-conjugated anti-rabbit IgG was applied as a secondary antibody and the positive bands were detected using Amersham ECL plus Western Blotting Detection Reagents (GE Health care, Piscataway, NJ).

#### Caspase 8 activity assay

MCF-7 cells were plated in 6-well plates and treated with DHA for different time periods. Then the cells were harvested and caspase 8 activity was measured by using a colorimetric ApoAlert Caspase-8 Assay Kit (colormetric) from Clonetech Laboratories, Inc. (Mountain View, CA) according to manufacturer's instructions.

### *In vivo* experiments

#### Experimental design

All procedures involving the use of live animals as described in this study were approved by the Institutional Animal Care and Use Committee of the University of Kansas Medical Center, and the investigators strictly followed the NIH guidelines for humane treatment of animals. Female athymic *nu/nu* mice, 4–5 weeks of age, were obtained from Harlan Laboratories (Indianapolis, IN). They were exposed to a 12-h light/12-h dark cycle, and had free access to a Harlan Teklad Rodent Diet 8604 (Harlan Teklad, Madison, WI) and water. Mice were housed under aseptic conditions (positive air pressure in a designated mouse room, with microisolator tops) and all mouse handling procedures were carried out under a laminar flow hood. After approximately one week of acclimatization after arrival, MCF-7 or MDA-MB-435s cells (5×10^6^ cells in 100 µL PBS) were s.c. injected into the right and left flanks of the animals. They were fed Harlan Teklad Rodent Diet 8604 while the injected cancer cells were allowed to grow for 2 weeks into tumors in the host animals before the start of feeding the experimental diets. The tumor-bearing mice were randomized into the following four groups (5 animals per group): Group 1 (MCF-7 cell xenograft), Group 2 (MCF-7 cell xenograft+fish oil diet), Group 3 (MDA-MB-435s cell xenograft), and Group 4 (MDA-MB-435s cell xenografts+fish oil diet). One of the groups of mice was treated with the control diet (AIN-76A diet containing 5% corn oil, Harlan Teklad., Madison, WI) and all other groups of animals were fed the fish oil-supplemented diet (AIN-76A diet containing 5% menhaden oil, prepared by Harlan Teklad, Madison, WI). These diets contained similar quantities of carbohydrates, protein, lipids, vitamins, and minerals (summarized in **Table 1**), and the only difference is the types of lipids (*i.e.*, corn oil *v*s. fish oil). Both diets were stored in sealed containers at −20°C to reduce spontaneous lipid peroxidation.

**Table 1:** Composition of the experimental diets used in this study.

	Control diet	Fish oil diet
Casein (g/Kg)	200.0	200.0
DL-Methionine (g/Kg)	3.0	3.0
Sucrose (g/Kg)	500.0	500.0
Corn starch (g/Kg)	150.0	150.0
Corn oil (g/Kg)	50.0^1^	-
Fish oil (g/Kg)	-	50.0^1^
Cellulose (g/Kg)	50.0	50.0
Mineral mix, AIN-76 (170915) (g/Kg)	35.0	35.0
Vitamin mix, AIN-76A (40077) (g/Kg)	10.0	10.0
Choline bitartrate (g/Kg)	2.0	2.0
Ethoxyquin, antioxidant (g/Kg)	0.01	0.01

1 Differs in diet.

#### Tumor and body weight measurements

The maximum and minimum diameters of the tumors (using a sliding caliper), body weight and amount of food intake were measured twice a week. Assuming that tumors formed in the animals were spherical, their volume was calculated using the formula [π/6×d^3^], where d is the mean diameter [20].

#### Analysis of plasma and tissue FA levels

Plasma and tissues (breast, uterus, skin, and tumor) were harvested from each animal, snap-frozen in liquid nitrogen, and stored in a −80°C freezer until analysis. The measurement of linoleic acid, arachidonic acid, EPA and DHA in plasma and tissues were conducted as described earlier [21]–[23]. Briefly, total lipids from plasma were extracted using the chloroform∶methanol mixture (2∶1, *v/v*), dried under a stream of nitrogen, and transmethylated using boron trifluoride in methanol (14 g/L). FA methyl esters were extracted from the mixture with pentane containing 0.05% butylated hydroxytoluene. One microliter of transmethylated sample was injected into the Agilent gas chromatography 6890N linked with the 5975B mass spectrometer. Capillary column HP5-MS (30 m×0.25 mm, 0.25 µm film thickness) was used for separation and helium as the carrier gas. The column oven temperature was set at 120°C, ramped to 250°C at 3°C/min, then ramped to 300°C at 10°C/min, and held at 300°C for 5 min.

#### Morphological and histopathological analyses

Tumor samples from each animal were removed and fixed in 10% buffered formalin phosphate (Fisher Scientific, Pittsburgh, PA), cryosectioned to 5-µm thickness, and stained with hematoxylin and eosin (H/E).

For immunohistochemical staining of the proliferating cell nuclear antigen (PCNA) and 3-nitrotyrosine, antigen retrieval procedure was performed by placing the slides in 10 mM citrate buffer (pH 3.0), heating in a microwave oven for 20 min and then allowing the slides to cool to room temperature for 20 min. Slides were rinsed once with PBS, and endogenous peroxidase activity was blocked by incubating the samples for 30 min with 3% H_2_O_2_ in PBS, followed by rinsing 3 times with PBS. Nonspecific binding was blocked by incubating the slides for 30 min in 2% normal goat serum (Vector Laboratories, Burlingame, CA) in 1% Triton X-100 containing PBS, followed by incubation with the specific antibodies against PCNA (1∶500 dilution, Abcam Inc., Cambridge, MA) and 3-nitrotyrosine (1∶100 dilution, Cell Signaling Technology Inc., Danvers, MA) in the blocking solution as described above. Then, slides were incubated with biotinylated goat anti-rabbit IgG (dilution, 1∶500, Vector Laboratories) in the blocking solution as described above. Next, the slides were incubated for 2 h with the avidin-biotin peroxidase complex (Vector Laboratories) according to the manufacturer's instructions, followed by 5 min incubation with a DAB substrate kit (Vector Laboratories). Counterstaining was performed using Mayer's hematoxylin. Negative controls lacking the primary antibody were used for each staining.

Apoptotic DNA degradation in tumor tissue was stained by the TUNEL method using the ApopTag® Plus Peroxidase *In Situ* Apoptosis Detection Kit (Chemicon International).

### Data analysis

The quantitative data were expressed as means ± S.D.. Statistical significance was determined using the analysis of variance (ANOVA) followed by a multiple comparison test with a Bonferroni adjustment. *P* values of <0.05 were considered statistically significant.

## Results

### DHA and EPA inhibit the growth of human breast cancer cells *in vitro*

**Figure 1:**
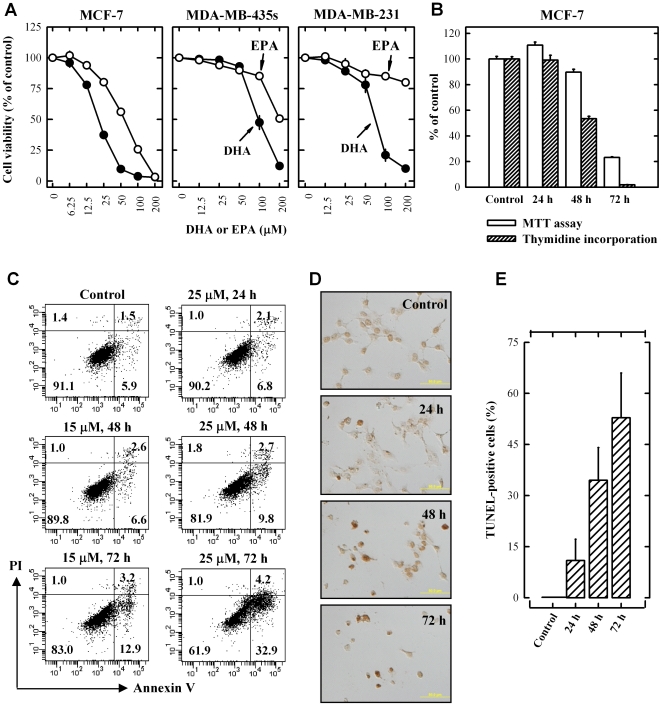
DHA and EPA inhibit MCF-7 cell growth in a concentration-dependent manner. (**A**) Cell viability (MTT assay) of MCF-7 cells, MDA-MB-231 and MDA-MB-435s cells treated with DHA or EPA at indicated concentrations for 72 h. Each value is the mean ± S.E. (n = 6). Note that for some of the data points, the error bars are smaller than the diameter of the symbols. (**B**) MTT and thymidine incorporation assays for MCF-7 cells treated with 25 µM DHA for the indicated length of time. Each value is the mean ± S.E. (n = 6 for both assays). (**C**) Annexin V/PI staining of MCF-7 cells treated with 15 µM and 25 µM DHA for the indicated length of time. The percentage of cells in each of the four quadrants was shown inside of each area. (**D**) TUNEL staining of MCF-7 cells treated with 25 µM DHA for the indicated length of time. The dark brown cells were positively-stained cells. (**E**) Quantification of TUNEL-positive cells as percentage of total cells. Each value is the mean ± S.D. (n = 3).

We first determined the effect of DHA and EPA on the viability (MTT assay) of cultured MCF-7, MDA-MB-231 and MDA-MB-435s cells. As shown in **Fig. 1A**, treatment with DHA and EPA for 3 days inhibited the viability of MCF-7 cells in a concentration-dependent manner, with IC_50_ values of 20.2 and 57.4 µM, respectively. Compared to MCF-7 cells, MDA-MB-435s and MDA-MB-231 cells were less sensitive to DHA and EPA, with IC_50_ values of approximately 70–100 µM and >200 µM, respectively. The time-dependent reduction of cell viability and inhibition of DNA synthesis in cultured MCF-7 cells following treatment with 25 µM DHA were compared by using the MTT assay and [^3^H]thymidine incorporation assay, respectively. As shown in **Fig. 1B**, cell viability (MTT assay) started to decrease at 48 h after DHA treatment, and approximately 80% of the cells lost viability at 72 h. In comparison, ^3^H-thymidine incorporation assay showed that when DNA synthesis was inhibited by 50% at 48 h, over 90% of cell viability was still retained, suggesting that inhibition of DNA synthesis might precede the reduction in cell viability.

To determine whether DHA (a representative omega-3 FA) also induced cell death, the annexin V/PI double staining method was used. With this method, cells stained single positive for PI were considered mostly necrotic cells, and cells stained single-positive for annexin-V were considered mostly early apoptotic cells, but cells that were stained double-positive could be either necrotic or apoptotic cells [24]–[26]. As shown in **Fig. 1C**, there was no increase in the PI single positive cells (reflecting mostly necrotic cells) after treatment of MCF-7 cells with 15 or 25 µM DHA at several time points tested. Instead, the annxin V single positive cells (reflecting mostly apoptotic cells) were increased following DHA treatment in a dose- and time-dependent manner. As a validation of the flow cytometry data on the DHA-induced apoptotic cell death, we also quantified the TUNEL-positive cells following DHA treatment of MCF-7 cells. As expected, the TUNEL-positive cells were increased after treatment with 25 µM DHA in a time-dependent manner (**Fig. 1D, 1E**). The percentage of TUNEL-positive cells correlated closely with the annexin V-positive cells in **Fig. 1C**. Taken together, the data from both annexin V-PI double staining and TUNEL assay consistently showed that DHA predominantly induced apoptosis in cultured MCF-7 cells.

### DHA-induced death in MCF-7 cells is mediated via caspase activation

**Figure 2:**
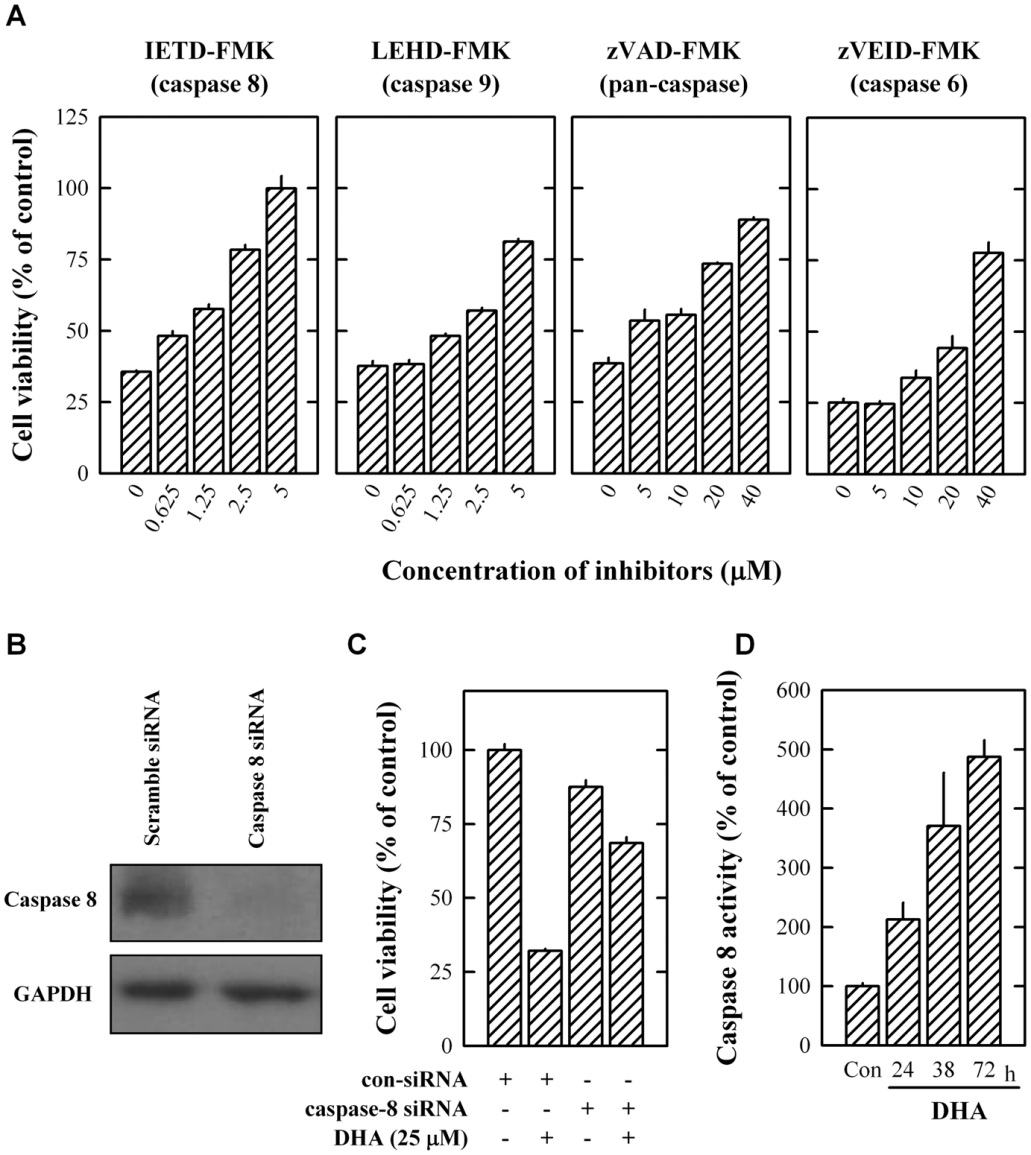
DHA-induced death of MCF-7 cells is mediated by caspase activation. (**A**) The protective effect of caspase inhibitors on 25 µM DHA-induced cell viability change of cultured MCF-7 cells. Cells were treated with DHA in the presence of different concentrations of the caspase inhibitor IETD-FMK (for caspase 8), LEHD-FMK (for caspase 9), zVAD-FMK (for caspases 1, 3, 4, 7), or zVEID-FMK (for caspase 6) for 72 h. Each value is the mean ± S.E. (n = 6). (**B**) Pro-caspase 8 protein expression level (Western blot analysis) in MCF-7 cells after transfection with scramble or caspase 8-specific siRNA (without DHA treatment). (**C**) The effect of caspase 8 knockdown on 25 µM DHA-induced death of cultured MCF-7 cells. Cells were first transfected with scramble or caspase 8-specific siRNA, and then they were treated with 25 µM DHA for additional 72 h. Each value is the mean ± S.E. (n = 6). (**D**) Time-dependent increase of caspase 8 activity in MCF-7 cells treated with 25 µM DHA for 24, 48, or 72 h. Each value is the mean ± S.E. (n = 3).

To determine whether activation of caspases was involved in DHA-induced death in cultured MCF-7 cells, pharmacological inhibitors of caspases were employed to probe whether they could protect cells from undergoing apoptosis. When the inhibitor of either caspase 8 or 9 was present, each of them strongly protected, in a concentration-dependent manner the death induced by 25 µM DHA. The protective effect of the caspase 8 inhibitor (IETD-FMK) was stronger than that of the caspase 9 inhibitor (LEHD-FMK). For instance, the caspase 8 inhibitor at 5 µM concentration completely abrogated DHA-induced loss of cell viability in MCF-7 cells whereas the caspase 9 inhibitor restored approximately 80% of cell viability. When the pan-caspase inhibitor zVAD-FMK (an inhibititor of caspases 1, 3, 4 and 7), or the caspase 6 inhibitor (zVEID-FMK) was present, a weaker protection against DHA-induced cell death was observed (**Fig. 2A**). To confirm the crucial role of caspase 8 in mediating DHA-induced death in MCF-7 cells, we selectively knocked down its expression in these cells using the caspase 8-specific siRNA. Western blotting of caspase 8 confirmed that procaspase 8 expression in caspase 8 siRNA-transfected cells was markedly decreased (**Fig. 2B**). As expected, knockdown of caspase 8 almost completely abolished the apoptotic effect of DHA (**Fig. 2C**). To confirm that DHA treatment results in activation of caspase 8, we determined its catalytic activity with a colormetric kit. As shown in **Fig. 2D**, caspase 8 activity was increased up to 5-fold after DHA treatment at 72 h.

Taken together, these data demonstrated that caspase 8 plays a critical role in mediating DHA-induced apoptotic cell death. The observation that caspase 8 inhibition can completely block DHA-induced cell death, in turn, provides support for the suggestion that apoptosis is the predominant form of cell death induced by DHA.

### DHA induces ROS formation in MCF-7 cells

**Figure 3:**
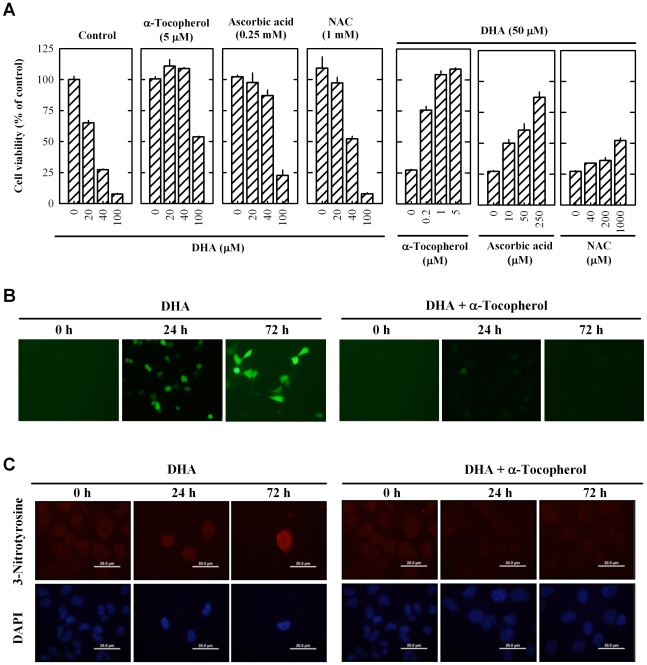
DHA induces ROS accumulation in MCF-7 cells. (**A**) Cell viability (MTT assay) of MCF-7 cells treated with different concentrations of α-tocoperol, ascorbic acid, or N-acetylcysteine (NAC) and DHA. Each value is the mean ± S.E. (n = 6). (**B**) ROS accumulation measured by H_2_-DCF-DA staining of MCF-7 cells treated with 25 µM DHA or together with 1 µM α-tocoperol for indicated time periods. The pictures were taken under a fluorescent microscope at the 100× magnification. (**C**) 3-Nitrotyrosine staining of MCF-7 cells treated with 25 µM DHA or together with 1 µM α-tocoperol for indicated time periods. The pictures were taken under a fluorescent microscope at the 400× magnification. Each value is the mean ± S.E. (n = 3).

As shown in **Fig. 3A** **(left panel)**, the antioxidants α-tocopherol (vitamin E), ascorbic acid (vitamin C), and *N*-acetyl cysteine (NAC) each protected MCF-7 cells against DHA-induced death. α-Tocopherol was much more potent and efficacious than ascorbic acid and NAC in protecting MCF-7 cells against DHA-induced death (**Fig. 3A**, **right panel**). Using the H_2_-DCF-DA staining, intracellular ROS accumulation was readily detected in MCF-7 cells at 24 and 72 h following DHA treatment (as shown by the green fluorescence in **Fig. 3B**). Co-treatment of cells with 1 µM α-tocopherol completely abrogated ROS accumulation induced by 25 µM DHA, which supported the suggestion that DHA-induced apoptosis was initiated by ROS accumulation. As shown in **Fig. 3C**, 3-nitrotyrosine-positive cells were detected in DHA-treated cells at 24 to 72 h, indicating that ROS and reactive nitrogen species generated following DHA treatment caused oxidative protein modifications in these cells. In comparison, cells co-treated with α-tocopherol and DHA had no appreciable 3-nitrotyrosine staining.

### Fish oil-supplemented diet suppresses the growth of MCF-7 human breast cancer cells *in vivo*

**Figure 4:**
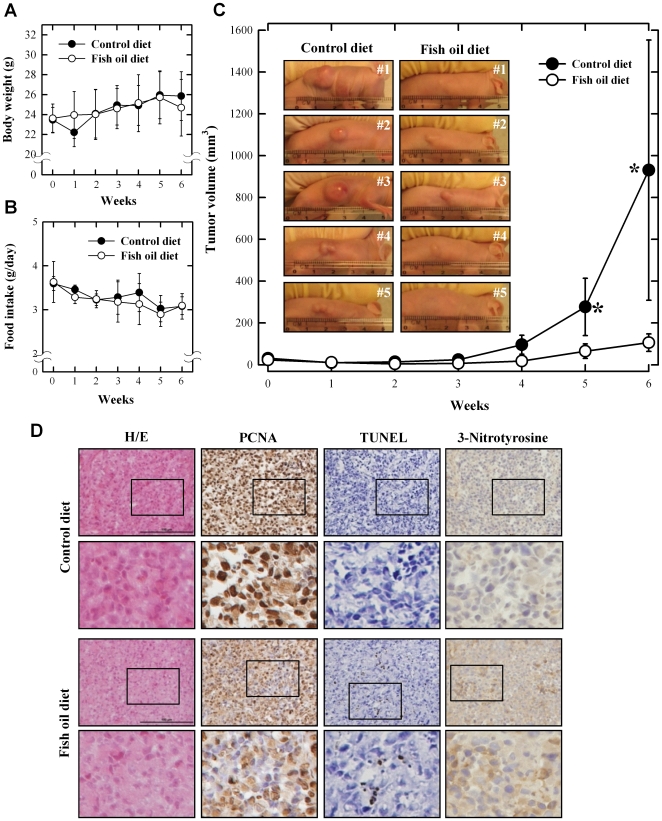
Fish oil-supplemented diet inhibits the growth of MCF-7 cancer cell xenografts in athymic nude mice. The changes in body weight (**A**), food intake amount (**B**) and tumor growth (**C**). The macroscopic pictures of tumor (**C**) and morphological and histopathological analyses (**D**) were assessed at the end of experiments. Nude mice were maintained on control or fish oil diet for 10 weeks after subcutaneous implantation of MCF-7 cells (5×10^6^ cells in 100 µL PBS). Both maximum and minimum diameters of the tumors, body weight and food intake amounts were measured twice a week using slide caliper. Tumor volume was calculated assuming the tumor to be spherical using the formula [π/6×d^3^], where d is the mean diameter.

To further assess the anticancer efficacy of omega-3 FAs *in vivo*, we used the cancer xenograft model in athymic nude mice. The animals were injected s.c. into their right and left flanks MCF-7 human breast cancer cells (at 5×10^6^ cells in 100 µL PBS), and two weeks later they were started to be fed a diet supplemented with 5% fish oil for 6 weeks. The average animal body weight was 23.5 g at the beginning of the fish oil diet treatment, and no significant differences in the body weight were seen among different treatment groups at the end of the experiment (**Fig. 4A**). Also, the amount of food intake between the control and fish oil diet groups was not significantly different throughout the experiment (**Fig. 4B**). However, significantly larger tumor size was observed in mice receiving the control diet (**Fig. 4C**), whereas in mice treated with the fish oil-supplemented diet their tumor size was basically not increased over the 6-week period (**Fig. 4C**).

Histological examination (H/E staining) of dissected tumor sections showed that the tumor cell density was reduced in fish oil diet-fed mice compared to the control diet group (**Fig. 4D**). PCNA-labeling indices of tumors from mice treated with the control diet were significantly higher than tumors from mice on the fish oil diet, suggesting that there was a decrease of cancer cell proliferation in the fish oil diet group. While the apoptotic indices (TUNEL assay) in tumor tissue of control diet-fed animals were mostly negative, the indices were markedly increased in animals fed a fish oil-supplemented diet.

The cellular levels of 3-nitrotyrosine, a commonly-used marker for protein nitration and cellular oxidative stress, were also determined in tumor xenografts as a parameter for the intracellular ROS levels. We found that the 3-nitrotyrosine level was significantly elevated in tumor tissues from fish oil diet-fed animals compared to control diet-fed animals (**Fig. 4D**).

**Figure 5:**
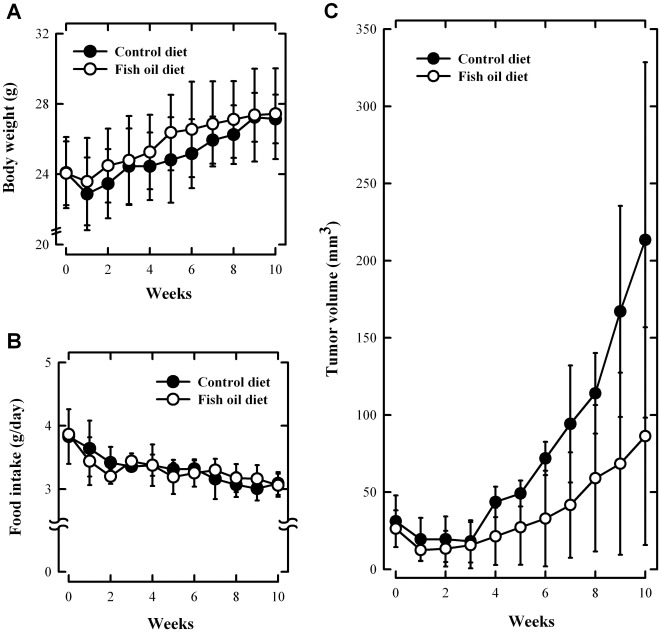
Fish oil-supplemented diet only slightly inhibits the growth of MDA-MB-435s cancer cell xenografts in athymic nude mice. The changes in body weight (**A**), food intake amount (**B**) and tumor growth (**C**). Nude mice were maintained on control or fish oil diet for 6 weeks after subcutaneous implantation of MDA-MB-435s cells (5×10^6^ cells in 100 µL PBS). Both maximum and minimum diameters of the tumors, body weight and food intake amounts were measured twice a week using slide caliper. Tumor volume was calculated assuming the tumor to be spherical using the following formula: **volume = π/6×** ***d*** **^3^**, where *d* is the mean diameter. Each value is the mean ± S.D. (n = 5).

Using the same tumor cell xenograft model, we also determined the effect of fish oil on the growth of MDA-MB-435s cells in nude mice for 10 weeks. As shown in **Fig. 5C**, the tumor volume in animals fed a fish oil-supplemented diet was only slightly smaller than that of the control diet group (but no statistical significance). Similar to what was observed with the MCF-7 cell xenograft experiment, animal body weight and food intake between the control and fish oil diet groups were not significantly different throughout the experiment (**Fig. 5A, 5B**). The lack of a significant anticancer effect of the fish oil diet in MDA-MB-435s tumor xenografts was consistent with the markedly weaker effect seen *in vitro* with this human cancer cell line. These data suggest that there was a cancer cell type-dependent sensitivity toward omega-3 FAs.

### Concentrations of FAs in diet, plasma and tissues

**Figure 6:**
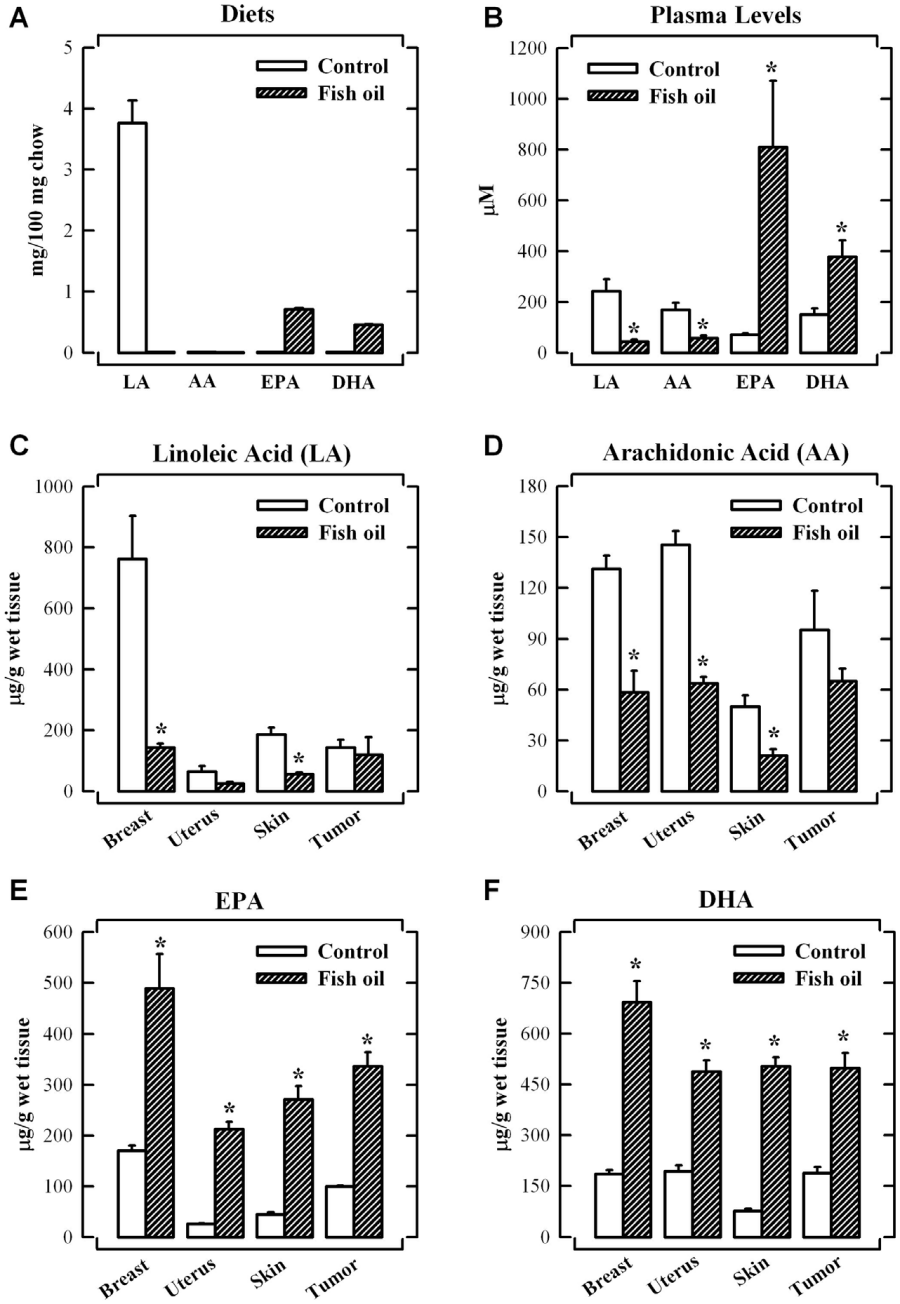
Omega-3 fatty acid levels in diets, plasma and tissues. Concentrations of FAs in food (**A**) and plasma (**B**). Comparison of the concentrations of LA (**C**), AA (**D**), EPA (**E**) and DHA (**F**) in various tissues. Nude mice were maintained on control or fish oil diet for 6 weeks after the subcutaneous implantation of MCF-7 cells (5×10^6^ cells in 100 µL PBS). Each value is the mean ± S.D. (n = 5). * *P*<0.05 *vs.* the control diet group.

The concentrations of linoleic acid, arachidonic acid, EPA and DHA in two different diets are shown in **Fig. 6A**. The control diet mainly consisted of linoleic acid (at 3.7±0.4 mg/100 mg diet), with negligible amount of arachidonic acid. In comparison, the fish oil-supplemented diet contained EPA and DHA at 0.7±0.1 and 0.5±0.0 mg/100 mg diet, respectively, whereas the levels of linoleic acid (LA) and arachidonic acid (AA) were below the detection limit.

The plasma levels of essential FAs were markedly changed following the 6-week feeding of the fish oil diet. While the omega-6 FA (linoleic acid and arachidonic acid) concentrations were higher than the omega-3 FAs (EPA and DHA) in the control diet group, it was reversed in the fish oil diet group (**Fig. 6B**). The animals fed the fish oil diet had significantly increased plasma levels of DHA and EPA by 2.5- and 11.3-fold, respectively, over those of the control group. Because EPA and DHA were basically undetectable in the control diet, their detection in the plasma of control diet-fed animals suggests that small amount of EPA and DHA can be biosynthesized in the body.

As shown in **Fig. 6C**, the amount of linoleic acid in the mammary tissue was highest among the tissues tested. However, the amount of linoleic acids in mammary, uterus and skin tissues were reduced to 5, 39 and 37% of the baseline levels, respectively, after feeding the fish oil diet (**Fig. 6C**). The tissue levels of arachidonic acid in the normal breast, uterus, skin and tumor tissues were 131.3±7.9, 145.6±8.0, 50.1±6.4 and 95.1±23.3 µg/g wet tissue, respectively, but they were markedly reduced in the fish oil diet group (**Fig. 6D**). The amount of linoleic acid and arachidonic acid in tumor tissues was not significantly changed by feeding the 5% fish oil diet (**Fig. 6C, 6D**). In the cases of omega-3 FAs, the levels of EPA and DHA in the tissues of control animals were less than 200 µg/g wet tissue, but they were significantly increased in all tissues after feeding 5% fish oil diet (**Fig. 6E, 6F**). Importantly, the change in ratio of omega-6 FAs (linoleic acid and arachidonic acid) to omega-3 FAs (EPA and DHA) in mammary tissue before and after fish oil diet treatment was more pronounced when compared to other tissues tested in this study.

## Discussion

The results of our present study showed that DHA, an omega-3 FA, has a strong anticancer activity in cultured MCF-7 human breast cancer cells through a combination of multiple actions, including inhibition of DNA synthesis, suppression of cell viability, and induction of apoptotic cell death. The strong *in vitro* anticancer effect was also observed *in vivo* in the athymic nude mouse model. These data confirmed and extended earlier observations [17]–[19], [27]–[29] on the anticancer effect of omega-3 FAs.

Mechanistically, the cell death induced by DHA is largely mediated through increased production of ROS in cancer cells, which subsequently results in apoptotic cell death. The crucial role of ROS in DHA-induced death of MCF-7 cells is supported by the following lines of observations made in the present study: First, there was an increase in the intracellular ROS accumulation and protein nitration in MCF-7 cells following treatment with DHA (**Fig. 3B, 3C**). Second, the cytotoxicity induced by DHA in MCF-7 cells was effectively protected by the co-presence of antioxidants (**Fig. 3A**). Third, based on the cellular levels of 3-nitrotyrosine as a marker for protein nitration and cellular oxidative stress, we also demonstrated that there was a marked increase in 3-nitrotyrosine levels in tumor tissues in the fish oil diet-fed animals compared to the control diet-fed animals (**Fig. 4D**). This *in vivo* observation agreed well with the *in vitro* data showing that there was a marked increase in the oxidative stress in cells treated with DHA. Taken together, these data suggest that DHA-induced cell death is largely mediated through the formation of intracellular ROS. The correlation between the *in vitro* and *in vivo* observations adds weight to this mechanistic explanation.

It was reported earlier that while omega-3 FAs could selectively inhibit tumor cell proliferation, they were significantly less cytotoxic in normal cells [30], [31]. The results of our present study showed that the anticancer effect of DHA and EPA in different human cancer cell lines (such as MCF-7, MDA-MB-231 and MDA-MB-435s) was also markedly different (**Fig. 1A**). It was suggested earlier that the levels of superoxide dismutase 1 expression plays an important role in determining the sensitivity of different tumor cells to the cytotoxic effects of DHA [32]. This suggestion appears to be in line with observations that increased oxidative stress contributes importantly to the anticancer actions of DHA.

A number of earlier studies have suggested that the anticancer property of DHA is attributable to its ability to induce apoptosis [17], [19], [27]–[29]. It was also reported that treatment of HL-60 cells with EPA results in caspases 3, 6, 8 and 9 activation, Bid cleavage, and cytochorme c release [33]. The results of our present study using annexin-V/PI double staining and TUNEL assay confirmed that DHA can induce apoptosis both *in vitro* and *in vivo* (**Fig. 1C, 1D, 1E** and **4D**). In addition, we found that caspase 8 is activated in a time-dependent manner, and either pharmacological inhibition or knockdown of caspase 8 almost completely protects cells from DHA-induced cell death (**Fig. 2**). Although pharmacological inhibition of caspase 9 also exerts a strong protection, its protective effect is not as strong as caspase 8 inhibition. Because caspase 9 is known to be activated following caspase 8 activation in MCF-7 cells, which are Type II cells [34], it is postulated that caspase 8 activation is an initiating event in DHA-induced apoptotic cell death.

**Figure 7:**
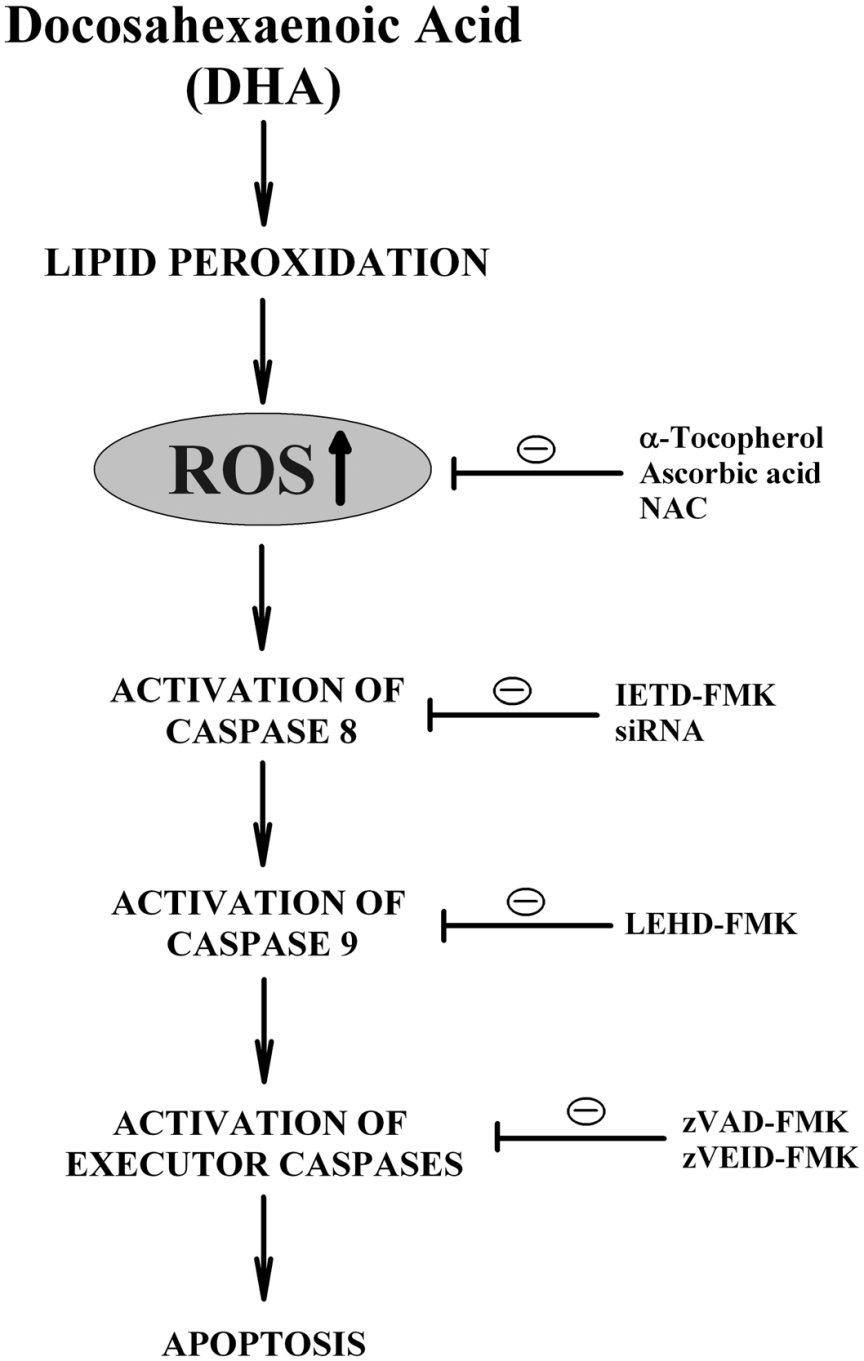
A scheme depicting the mechanism of DHA-induced death in MCF-7 human breast cancer cells. The presence of markedly elevated intracellular levels of DHA would increase lipid peroxidation and result in increased production of ROS. Accumulation of ROS results in the activation of caspase 8, which then activates its downstream caspase 9 as well as executor caspases. The activation of these caspases results in the induction of apoptotic cell death. When antioxidants are co-present, they can effectively protect cells from undergoing apoptosis by suppressing ROS formation. In comparison, the presence of inhibitors for various caspases or the selective knockdown of caspase 8 each shows a strong protection of cells from DHA-induced apoptosis.

The mechanism by which DHA selectively activates caspase 8 is still not understood at present. It is speculated that omega-3 FAs may preferentially increase ROS accumulation in the plasma membrane lipid rafts where the assembly of the death-inducing signaling complex (DISC) and the subsequent activation of caspase 8 takes place [35]. The observation that the lipid-soluble α-tocopherol is more potent and efficacious in protecting DHA-induced apoptosis as compared to the water-soluble ascorbic acid or *N*-acetylcysteine (NAC) provides partial support for the notion that the production and accumulation of ROS in the lipid-rich micro-environments may contribute importantly to caspase 8 activation and, subsequently, apoptosis induction in DHA-treated cells. The proposed mechanism of DHA-induced apoptosis as observed in cultured MCF-7 human breast cancer cells is depicted in **Fig. 7**.

It is known that the membrane FA composition in cancer cells can be modified either in culture or during growth *in vivo* without disrupting the membrane integrity [36]. As shown in **Figure 6**, the levels of DHA in plasma and tissues (including mammary tissues and tumors) in animals fed a 5% fish oil diet are increased significantly. The total plasma concentrations of DHA and EPA in animals fed a diet supplemented with fish oil are approximately 400 µM and 800 µM, respectively, although it is not known what percentage of the detected omega-3 FAs is in the free (unconjugated) form. Notably, while the concentrations of linoleic acid (the source of arachidonic acid) in animals fed a regular diet are highest in breast tissue compared to a few other tissues examined in this study, feeding animals with a diet supplemented with fish oil for 6 weeks completely alters the composition of various fatty acids in breast tissue, with a marked increase in EPA and DHA levels along with a drastic decrease in linoleic acid levels. Since a strong suppression of tumor growth is seen in animals fed a fish oil-supplemented diet while no significant change of animal body weight (or other signs of adverse effects) is observed, it is believed that effective concentrations of omega-3 FAs likely are achievable *in vivo* following oral ingestion of omega-3 FAs. Also, it is apparent that the omega-3 FAs are essentially non-toxic *in vivo* at the effective anticancer dose used.

Epidemiological studies have led to the suggestion that at least one-third of the human cancers are associated with diet [37]. It was estimated that an adult human could consume approximately 10–12 g fish oil per day without noticeable adverse effects [38]. Based on this information, the effective dose used in the present preclinical study through feeding animals a 5% fish oil-supplemented diet may also be achievable in humans through increased dietary intake [39]. The results of our present study, along with the data from recent studies by others suggest that DHA may improve the outcomes of chemotherapy in breast cancer patients via increased ROS formation [40], [41], call for additional studies to determine whether the use of dietary omega-3 FAs will have a similar suppressive effect on cancer growth in breast cancer patients.
